# Development and validation of a risk prediction model for osteoporosis in elderly patients with type 2 diabetes mellitus: a retrospective and multicenter study

**DOI:** 10.1186/s12877-023-04306-1

**Published:** 2023-10-27

**Authors:** Juntao Tan, Zhengyu Zhang, Yuxin He, Xiaomei Xu, Yanzhi Yang, Qian Xu, Yuan Yuan, Xin Wu, Jianhua Niu, Songjia Tang, Xiaoxin Wu, Yongjun Hu

**Affiliations:** 1 Operation Management Office, Affiliated Banan Hospital of Chongqing Medical University, Chongqing, 401320 China; 2 Medical Records Department, the First Affiliated Hospital, College of Medicine, Zhejiang University, Hangzhou, 310003 China; 3 Department of Medical Administration, Affiliated Banan Hospital of Chongqing Medical University, Chongqing, 401320 China; 4 Department of Infectious Diseases, Chengdu Fifth People’s hospital, Chengdu, 611130 China; 5 Department of Endocrinology and Metabolism, Chengdu First People’s Hospital, Chengdu, 610041 China; 6 College of Medical Informatics, Chongqing Medical University, Chongqing, 400016 China; 7 Medical Data Science Academy, Chongqing Medical University, Chongqing, 400016 China; 8 Library, Chongqing Medical University, Chongqing, 400016 China; 9 Medical Records Department, Women and Children’s Hospital of Chongqing Medical University, Chongqing, 401147 China; 10 Department of Gastrointestinal surgery, Third Affiliated Hospital of Chongqing Medical University, Chongqing, 401120 China; 11 Department of Critical Care, the First Affiliated Hospital, Zhejiang University School of Medicine, 79 Qing Chun Road, Hangzhou, 310003 Zhejiang China; 12 Plastic and Aesthetic Surgery Department, Affiliated Hangzhou First People’s Hospital, Zhejiang University School of Medicine, Hangzhou, 310000 Zhejiang China; 13 State Key Laboratory for Diagnosis and Treatment of Infectious Diseases, National Clinical Research Centre for Infectious Diseases, the First Affiliated Hospital, Zhejiang University School of Medicine, 79 Qing Chun Road, Hangzhou, 310003 Zhejiang China; 14 Department of Orthopedics, Affiliated Banan Hospital of Chongqing Medical University, Chongqing, 401320 China

**Keywords:** Type 2 diabetes mellitus, Osteoporosis, Elderly patients, Prediction model

## Abstract

**Background:**

This study aimed to construct a risk prediction model to estimate the odds of osteoporosis (OP) in elderly patients with type 2 diabetes mellitus (T2DM) and evaluate its prediction efficiency.

**Methods:**

This study included 21,070 elderly patients with T2DM who were hospitalized at six tertiary hospitals in Southwest China between 2012 and 2022. Univariate logistic regression analysis was used to screen for potential influencing factors of OP and least absolute shrinkage. Further, selection operator regression (LASSO) and multivariate logistic regression analyses were performed to select variables for developing a novel predictive model. The area under the receiver operating characteristic curve (AUROC), calibration curve, decision curve analysis (DCA), and clinical impact curve (CIC) were used to evaluate the performance and clinical utility of the model.

**Results:**

The incidence of OP in elderly patients with T2DM was 7.01% (1,476/21,070). Age, sex, hypertension, coronary heart disease, cerebral infarction, hyperlipidemia, and surgical history were the influencing factors. The seven-variable model displayed an AUROC of 0.713 (95% confidence interval [CI]:0.697–0.730) in the training set, 0.716 (95% CI: 0.691–0.740) in the internal validation set, and 0.694 (95% CI: 0.653–0.735) in the external validation set. The optimal decision probability cut-off value was 0.075. The calibration curve (bootstrap = 1,000) showed good calibration. In addition, the DCA and CIC demonstrated good clinical practicality. An operating interface on a webpage (https://juntaotan.shinyapps.io/osteoporosis/) was developed to provide convenient access for users.

**Conclusions:**

This study constructed a highly accurate model to predict OP in elderly patients with T2DM. This model incorporates demographic characteristics and clinical risk factors and may be easily used to facilitate individualized prediction.

**Supplementary Information:**

The online version contains supplementary material available at 10.1186/s12877-023-04306-1.

## Introduction

Osteoporosis (OP) is a clinically common systemic bone disease that increases the risk of brittle fractures due to reduced bone mass and the breakdown of the bone tissue microstructure [1, 2]. Approximately 200 million people worldwide while approximately 88 million people in China suffer from osteoporosis [3]. Under the trend of global population aging, OP becomes increasingly widespread [4]. Recent studies indicate that elderly patients with type 2 diabetes mellitus (T2DM) have a high incidence of OP, which affects their quality of life and leads to high disability and mortality rates [5–7]. A recent meta-analysis of 21 studies involving 11,603 T2DM patients found a high OP prevalence of 27.67% (95% confidence interval (CI) 21.37–33.98%) [8].

At present, bone mineral density (BMD) testing is the main method for OP screening or diagnosing, as it is a strong and consistent predictor of OP. A single measure of BMD can predict OP risk over 25 years, with little degradation in this association over time [9]. In addition, there are other OP screening tools such as the fracture risk assessment tool (FRAX) [10], the male osteoporosis risk estimation score (MORES) [11], and the osteoporosis self-assessment tool for Asians (OSTA) [12].

However, the pathogenesis of OP in elderly patients with T2DM remains ambiguous. In addition to factors related to age, sex, race, and genetics, poor blood sugar control in T2DM patients leads to osmotic diuresis, and a large amount of calcium ions is lost from the urine, which leads to abnormal metabolism of vitamin D and parathyroid hormone. Ultimately this results in abnormal bone metabolism [13, 14]. Secondly, poor long-term blood glucose control leads to an increase in advanced glycation end products, which ends in the abnormal metabolism of bone organic matter, weakened osteogenesis, and enhanced osteoclasis, ultimately causing a high incidence of OP in diabetes patients [15].

OP diagnosis is relatively delayed and is prone to brittle fractures. Additionally, patients do not receive early prevention and treatment. Therefore, an early diagnosis plays a decisive role in disease development and prognosis. In this novel study, we identified the factors influencing OP by analyzing the clinical characteristics of elderly patients with T2DM admitted to six tertiary hospitals in Southwest China, and developed a predictive risk model for OP. Furthermore, we sought to develop a user-friendly interface via a web link to calculate the precise probability of OP in elderly patients with T2DM. These tools were designed to support quality improvement and aid in the clinical management of elderly patients with T2DM.

## Materials and methods

### Data source

This was a retrospective multicenter study. The study followed the guidelines for transparent reporting of a multivariable prediction model for individual prognosis or diagnosis (TRIPOD) [16]. The clinical data of 21,070 elderly patients with T2DM were obtained from six tertiary hospitals in Southwest China from 2012 to 2022. Using a random number method, the “caret” R package, patients from hospitals A-E were randomly divided into a training set (n = 12,366) and an internal validation set (n = 5,301) at a ratio of 7:3. Patient data from hospital F were collected for external validation (n = 3,403). The study protocol was reviewed and approved by the ethics committee of the Affiliated Banan Hospital of Chongqing Medical University. Informed consent was not required because of the retrospective nature of the study.

### Inclusion and exclusion criteria

The inclusion criteria were : (i) diagnosed with T2DM between 2012 and 2022, and (ii) aged 65 years or older. The exclusion criteria involve: (i) combined thyroiditis and hyperthyroidism; (ii) combined with other bone metabolic disorders, such as rickets, osteomalacia, and osteosclerosis; (iii) concomitant with severe mental illness; (iv) recipient of calcium, glucocorticoid, calcitonin, or other drugs that affect bone metabolism; and (v) patients with > 30% missing data (after meeting the inclusion criteria and the exclusion criteria i, ii, iii, and iv, patients still had variables with more than 30% of missing data). The selection process is illustrated in **supplementary figure ****S1**.

### Definition

Severe mental illness was defined as conditions presenting as psychosis, including schizophrenia, schizoaffective disorder, bipolar disorder, and other psychotic disorders [17].

Bone mineral density was measured using whole-body dual-energy X-ray absorptiometry (DXA). The detection sites included the lumbar spine (LS) 1–4, femoral neck, greater trochanter of the femur, inner femur, and Ward’s triangular area. OP was defined if the T-score ≤ -2.5SD, according to the WHO criteria (1994) [18]. In addition, OP was identified using computable phenotypes based on billing codes from the International Classification of Diseases, Tenth Revision, Clinical Modification (ICD-10-CM). The ICD-10-CM codes M80, M81, and M82 were associated with OP.

### Data Collection

Based on previous reports, a total of 26 candidate variables were selected to reflect OP [19–21]. We explored age, sex, hypertension, coronary heart disease (CHD), cerebral infarction (CI), hyperlipidemia, past surgical history (PSH), past medical history (PMH), smoking history, drinking history, systolic blood pressure (SBP), diastolic blood pressure (DBP), pulse, aspartate aminotransferase (AST), triglycerides (TGs), neutrophil-lymphocyte ratio (NLR), platelet-lymphocyte ratio (PLR), lymphocyte-monocyte ratio (LMR), neutrophil percentage-to-albumin ratio (NPAR), creatinine (CREA), uric acid (UA), low-density lipoprotein cholesterol (LDL-C), high-density lipoprotein cholesterol (HDL-C), glycated hemoglobin (HbA1c), and glomerular filtration rate (GFR).

### Statistical analyses

Statistical analyses were performed using the SPSS (version 22.0; IBM Corp., Armonk, NY, USA) and R software (version 4.0.2; R Core Team, Vienna, Austria). The Kolmogorov–Smirnov normality test was performed on all measurement data. Indicators conforming to normal distribution were described as mean ± standard deviation, and a t-test was adopted. Indicators that did not conform to normal distribution were described as median (M) and quartile interval (P25, P75), and the Mann-Whitney U test was used. The enumeration data were expressed in terms of frequency and rate and were tested using the χ^2^ test or Fisher’s exact test. We used the R multivariate imputation by chained equation package for missing data imputation in this study. For all statistical analyses, the significance was set at P < 0.05.

Univariate logistic regression analysis was employed to screen for potential influencing factors of OP, and the least absolute shrinkage and selection operator regression (LASSO) and multivariate logistic regression analyses were performed to further select variables for developing a novel predictive model. The area under the receiver operating characteristic curve (AUROC), calibration curve, decision curve analysis (DCA), and clinical impact curve (CIC) were used to evaluate the performance and clinical utility of the model.

## Results

### Patient characteristics

The Mann-Whitney U test revealed that there was no significant difference in several missing variables in the training and internal validation sets before and after multiple imputations (Table 1). Furthermore, there were no significant differences in any missing variables in the external validation set before and after multiple imputations (**Supplementary Table 1**). In total, 21,070 elderly patients with T2DM were included in this study. The incidence of OP in elderly patients with T2DM was 7.01% (1,476/21,070). Table 2 lists the baseline characteristics of patients in the training and internal validation sets.

**Table 1:** Comparison of continuous variables in the training and internal validation sets before and after multiple imputation

Variables	Before interpolation	After interpolation	*P* values
SBP(IQR, mmHg)	138.00(126.00,153.00)	139.00(126.00,153.00)	0.833
DBP(IQR, mmHg)	78.00(70.00,85.00)	78.00(70.00,86.00)	0.607
Pulse(IQR, bpm)	80.00(72.00,89.00)	80.00(72.00,89.00)	0.895
AST(IQR, IU/L)	20.00(16.00,27.00)	20.00(16.00,27.00)	0.864
ALT(IQR, IU/L)	18.00(13.00,26.90)	18.00(13.00,26.60)	0.622
TGs(IQR, mmol/l)	1.45(1.05,2.07)	1.42(1.04,2.03)	0.012
NLR(IQR)	3.14(2.14,5.13)	3.17(2.14,5.23)	0.263
PLR(IQR)	125.95(92.45,177.16)	126.63(92.73,178.67)	0.286
LMR(IQR)	3.87(2.57,5.50)	3.84(2.54,5.49)	0.256
NPAR(IQR, ml/g)	17.28(14.96,20.39)	17.31(14.96,20.47)	0.466
CREA(IQR, umol/l)	70.40(57.00,89.90)	70.40(57.00,90.00)	0.930
UA(IQR, umol/l)	320.00(257.00,396.00)	320.00(256.90,396.90)	0.952
LDL-C(IQR, mmol/l)	2.44(1.87,3.08)	2.42(1.86,3.05)	0.043
HDL-C(IQR, mmol/l)	1.12(0.93,1.35)	1.12(0.93,1.35)	0.854
HbA1c(IQR, %)	7.70(6.70,10.00)	7.69(6.65,9.90)	0.097
GFR(IQR, mL/min)	85.63(65.54,102.38)	85.60(65.43,102.41)	0.873

*SBP: systolic blood pressure; DBP: diastolic blood pressure; AST:aspartate aminotransferase; ALT: alanine aminotransferase; TGs: triglycerides; NLR: neutrophil-to-lymphocyte ratio; PLR: platelet-to-lymphocyte ratio; LMR: lymphocyte to monocyte ratio; NPAR: neutrophil percentage-to-albumin ratio; CREA: creatinine; UA: uric acid; LDL-C: low density lipoprotein cholesterol; HDL-C: high density lipoprotein cholesterol; HbA1c: glycated hemoglobin; GFR: glomerular filtration rate; IQR: interquartile range*

**Table 2:** Demographic and clinical characteristics of the training and internal validation sets

Variables	Total (N = 17,667)	Training set (N = 12,366)	Internal validation set (N = 5,301)	*P* values
Age(IQR, year)	73.00(68.00,78.00)	73.00(68.00,78.00)	73.00(68.00,78.00)	0.795
Sex(n, %)				0.426
Female	9529	6694	2835	
Male	8138	5672	2466	
Hypertension(n, %)				0.177
No	7470	5188	2282	
Yes	10,197	7178	3019	
CHD(n, %)				0.593
No	12,796	8942	3854	
Yes	4871	3424	1447	
CI(n, %)				0.267
No	14,609	10,200	4409	
Yes	3058	2166	892	
Hyperlipidemia(n, %)				0.889
No	15,251	10,672	4579	
Yes	2416	1694	722	
PSH(n, %)				0.673
No	8519	5950	2569	
Yes	9148	6416	2732	
PMH(n, %)				0.417
No	1813	1254	559	
Yes	15,854	11,112	4742	
Amoking history(n, %)				0.278
No	12,879	9044	3835	
Yes	4788	3322	1466	
Srinking history(n, %)				0.089
No	13,761	9675	4086	
Yes	3906	2691	1215	
SBP(IQR, mmHg)	139.00(126.00,153.00)	139.00(126.00,154.00)	139.00(126.00,153.00)	0.301
DBP(IQR, mmHg)	78.00(70.00,86.00)	78.00(70.00,85.00)	78.00(70.00,86.00)	0.586
pulse(IQR, bpm)	80.00(72.00,89.00)	80.00(72.00,89.00)	80.00(72.00,90.00)	0.330
AST(IQR, IU/L)	20.00(16.00,27.00)	20.00(16.00,27.00)	20.00(16.00,27.00)	0.311
ALT(IQR, IU/L)	18.00(13.00,26.60)	18.00(13.00,27.00)	18.00(13.00,26.00)	0.541
TGs(IQR, mmol/l)	1.42(1.04,2.03)	1.43(1.04,2.02)	1.41(1.05,2.04)	0.765
NLR(IQR)	3.17(2.14,5.23)	3.17(2.14,5.25)	3.18(2.14,5.16)	0.988
PLR(IQR)	126.63(92.73,178.67)	126.84(92.79,179.39)	126.05(92.51,176.98)	0.687
LMR(IQR)	3.84(2.54,5.49)	3.85(2.54,5.45)	3.84(2.55,5.57)	0.833
NPAR(IQR, ml/g)	17.31(14.96,20.47)	17.35(14.96,20.52)	17.23(14.96,20.37)	0.396
CREA(IQR, umol/l)	70.40(57.00,90.00)	70.30(57.00,90.20)	70.40(56.90,89.40)	0.372
UA(IQR, umol/l)	320.00(256.90,396.90)	321.05(257.50,397.48)	317.30(255.00,395.30)	0.106
LDL-C(IQR, mmol/l)	2.42(1.86,3.05)	2.42(1.86,3.04)	2.42(1.87,3.06)	0.896
HDL-C(IQR, mmol/l)	1.12(0.93,1.35)	1.12(0.93,1.35)	1.13(0.93,1.35)	0.479
HbA1c(IQR, %)	7.69(6.65,9.90)	7.70(6.6125,9.85)	7.69(6.70,10.00)	0.503
GFR(IQR, mL/min)	85.60(65.43,102.41)	85.51(65.12,102.45)	85.87(65.96,102.31)	0.392

*CHD: coronary heart disease; CI: cerebral infarction; PSH: past surgical history; PMH: past medical history ; SBP: systolic blood pressure; DBP: diastolic blood pressure; AST:aspartate aminotransferase; ALT: alanine aminotransferase; TGs: triglycerides; NLR: neutrophil-to-lymphocyte ratio; PLR: platelet-to-lymphocyte ratio; LMR: lymphocyte to monocyte ratio; NPAR: neutrophil percentage-to-albumin ratio; CREA: creatinine; UA: uric acid; LDL-C: low density lipoprotein cholesterol; HDL-C: high density lipoprotein cholesterol; HbA1c: glycated hemoglobin; GFR: glomerular filtration rate; IQR: interquartile range*

### Selection of predictors

Patients in the training set were divided into OP and non-OP groups. The following factors were significantly associated with OP in univariate analysis: age, sex, hypertension, CHD, CI, hyperlipidemia, PSH, PMH, smoking history, drinking history, SBP, pulse, AST, ALT, NLR, PLR, LMR, NPAR, CREA, HbA1c, and GFR (P < 0.05) (Table 3).

**Table 3:** Demographic and clinical characteristics associated with OP as assessed in the training set

Variables	OP (N = 912)	Non-OP (N = 1,1454)	*P* values
Age(IQR, year)	76.00(70.00,81.00)	73.00(68.00,78.00)	< 0.001
Sex(n, %)			< 0.001
Female	709	5985	
Male	203	5469	
Hypertension(n, %)			< 0.001
No	273	4915	
Yes	639	6539	
CHD(n, %)			< 0.001
No	542	8400	
Yes	370	3054	
CI(n, %)			< 0.001
No	649	9551	
Yes	263	1903	
Hyperlipidemia(n, %)			< 0.001
No	707	9965	
Yes	205	1489	
PSH(n, %)			< 0.001
No	361	5589	
Yes	551	5865	
PMH(n, %)			0.004
No	67	1187	
Yes	845	10,267	
Smoking history(n, %)			< 0.001
No	776	8268	
Yes	136	3186	
Drinking history(n, %)			< 0.001
No	798	8877	
Yes	114	2577	
SBP(IQR, mmHg)	137.00(125.00,150.00)	139.00(126.00,154.00)	0.001
DBP(IQR, mmHg)	77.00(70.00,84.00)	78.00(70.00,85.00)	0.091
Pulse(IQR, bpm)	78.00(71.00,86.00)	80.00(72.00,90.00)	< 0.001
AST(IQR, IU/L)	20.00(16.00,25.00)	20.00(16.00,27.00)	0.002
ALT(IQR, IU/L)	17.00(12.00,23.85)	18.00(13.00,27.00)	< 0.001
TGs(IQR, mmol/l)	1.44(1.06,2.01)	1.43(1.04,2.02)	0.639
NLR(IQR)	2.84(2.00,4.45)	3.19(2.15,5.31)	< 0.001
PLR(IQR)	122.22(92.34,167.48)	127.27(92.95,180.37)	0.011
LMR(IQR)	4.18(2.83,5.79)	3.82(2.51,5.42)	< 0.001
NPAR(IQR, ml/g)	17.00(14.65,20.03)	17.37(14.98,20.57)	0.004
CREA(IQR, umol/l)	66.85(54.50,88.50)	70.50(57.10,90.40)	< 0.001
UA(IQR, umol/l)	316.30(253.98,391.68)	321.5(257.93,397.98)	0.246
LDL-C(IQR, mmol/l)	2.39(1.85,3.04)	2.43(1.86,3.04)	0.534
HDL-C(IQR, mmol/l)	1.13(0.96,1.35)	1.12(0.92,1.35)	0.072
HbA1c(IQR, %)	7.40(6.60,9.70)	7.70(6.64,9.90)	0.048
GFR(IQR, mL/min)	82.71(60.22,95.81)	85.72(65.56,103.05)	< 0.001

*OP:osteoporosis; CHD: coronary heart disease; CI: cerebral infarction; PSH: past surgical history; PMH: past medical history ; SBP: systolic blood pressure; DBP: diastolic blood pressure; AST:aspartate aminotransferase; ALT: alanine aminotransferase; TGs: triglycerides; NLR: neutrophil-to-lymphocyte ratio; PLR: platelet-to-lymphocyte ratio; LMR: lymphocyte to monocyte ratio; NPAR: neutrophil percentage-to-albumin ratio; CREA: creatinine; UA: uric acid; LDL-C: low density lipoprotein cholesterol; HDL-C: high density lipoprotein cholesterol; HbA1c: glycated hemoglobin; GFR: glomerular filtration rate; IQR: interquartile range.*

**Fig. 1:**
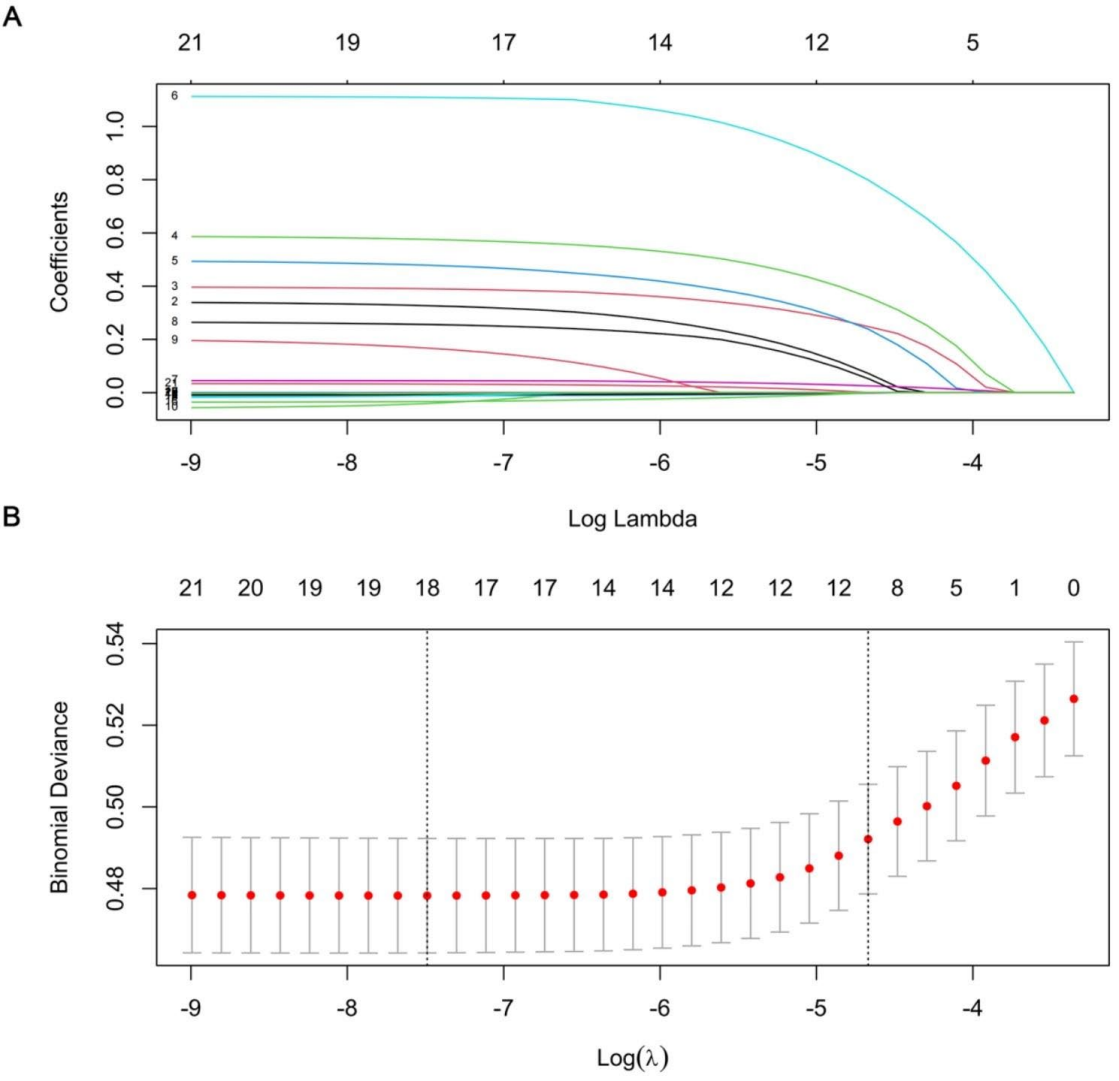
Features selection by LASSO. A LASSO coefcients profles (y-axis) of the 21 features. The upper x-axis is the average numbers of predictors and the lower x-axis is the log(λ). B Tenfold cross-validation for tuning parameter selection in the LASSO model

**Fig. 2:**
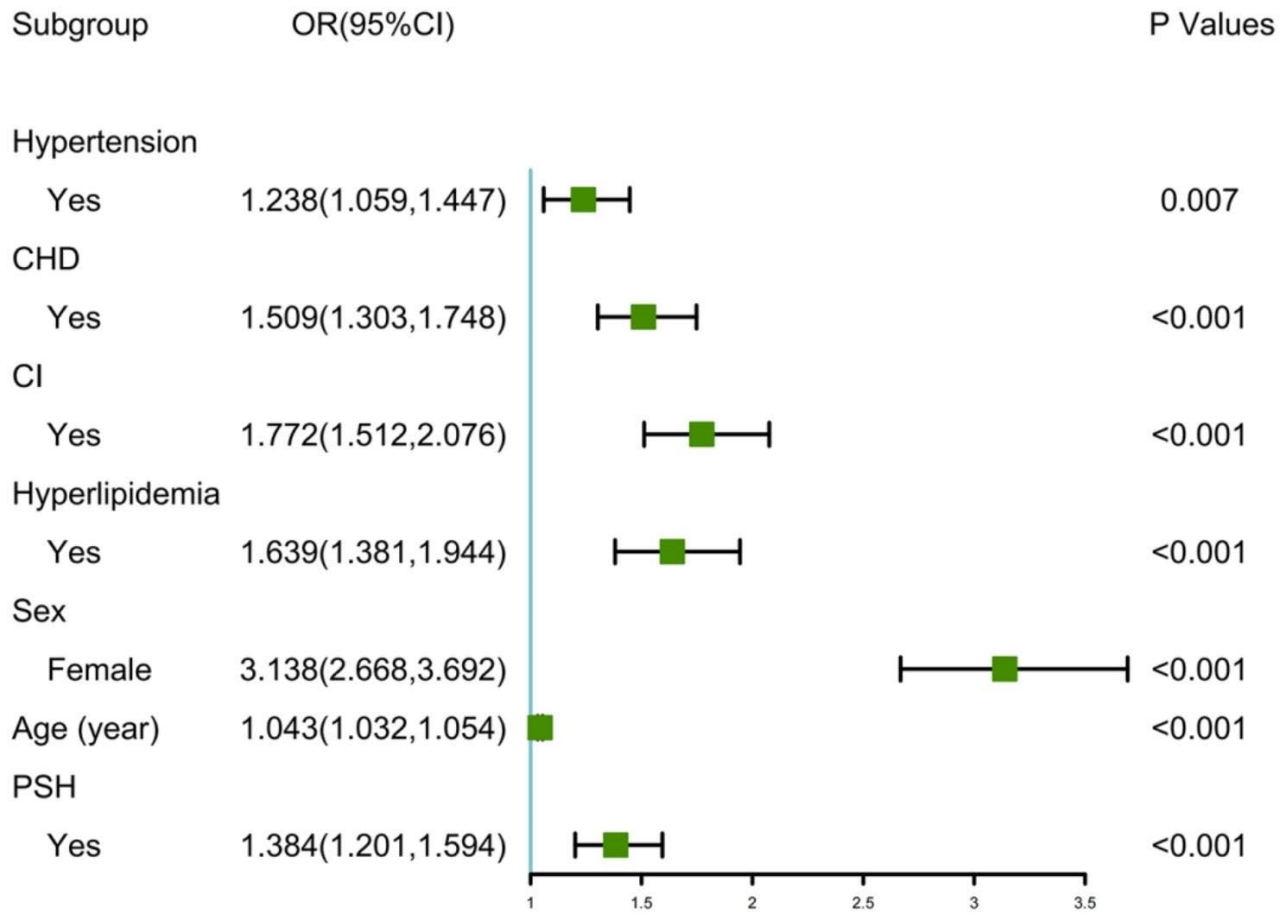
Forest plot showing the results of multivariable analysis for OP

As depicted in Fig. 1, the model chose Lambda corresponding to a value of 0.009374144 and selected 11 predictors: age, sex, hypertension, CHD, CI, hyperlipidemia, PSH, SBP, pulse, NLR, and HbA1c. Ultimately, the multivariate logistic regression model depicted that age (odds ratio [OR] = 1.043, 95% confidence interval [CI]:1.032–1.054), sex (OR = 3.138, 95% CI: 2.668–3.692), hypertension (OR = 1.238, 95% CI: 1.059–1.447), CHD (OR = 1.509, 95% CI: 1.303–1.748), CI (OR = 1.772, 95% CI: 1.512–2.076), hyperlipidemia (OR = 1.639, 95% CI: 1.381–1.944), and PSH (OR = 1.384, 95% CI: 1.201–1.594) were the influencing factors for predicting OP (Fig. 2).

**Fig. 3:**
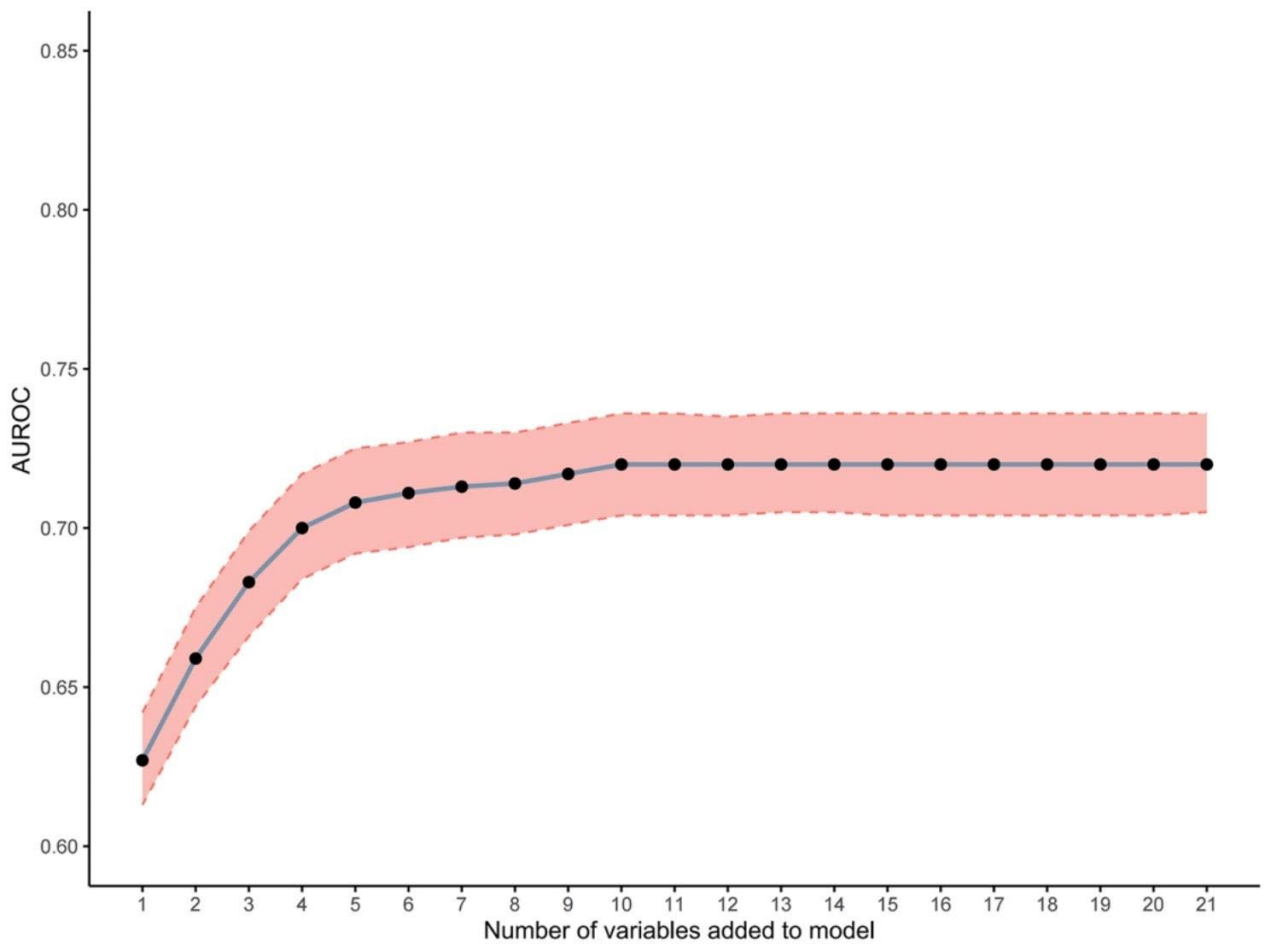
Identification of the optimal variables numbers for a prediction of OP

To further validate the performance of LASSO-logistic regression in screening predictive variables, we evaluated variable subsets with the top k features, k ranging between 1 and 21, to identify the threshold at which adding variables to the predictive model would not significantly improve its performance. Finally, we identified seven variables with the highest information gain and found no significant increase in the AUROC after including such variables (AUROC = 0.713, P = 0.134, Fig. 3), which were consistent with the variables in the LASSO-logistic regression model. This finding indicates that adding more variables, even those closely related to OP, may not necessarily improve model performance (mean rolling P value for the remaining variable sets: 0.404).

### Nomogram prediction model construction and performance

**Fig. 4:**
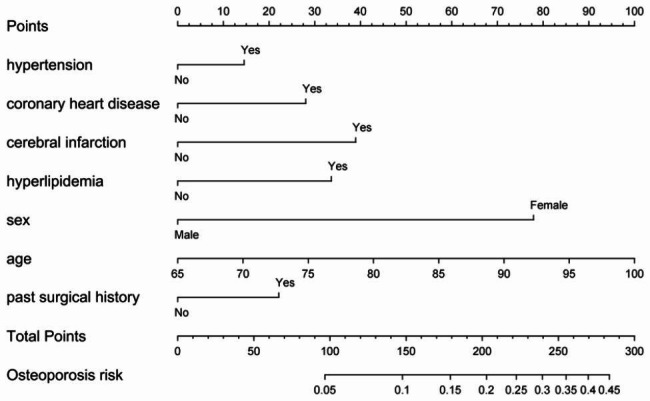
Nomogram predicting OP in elderly patients with T2DM

**Fig. 5:**
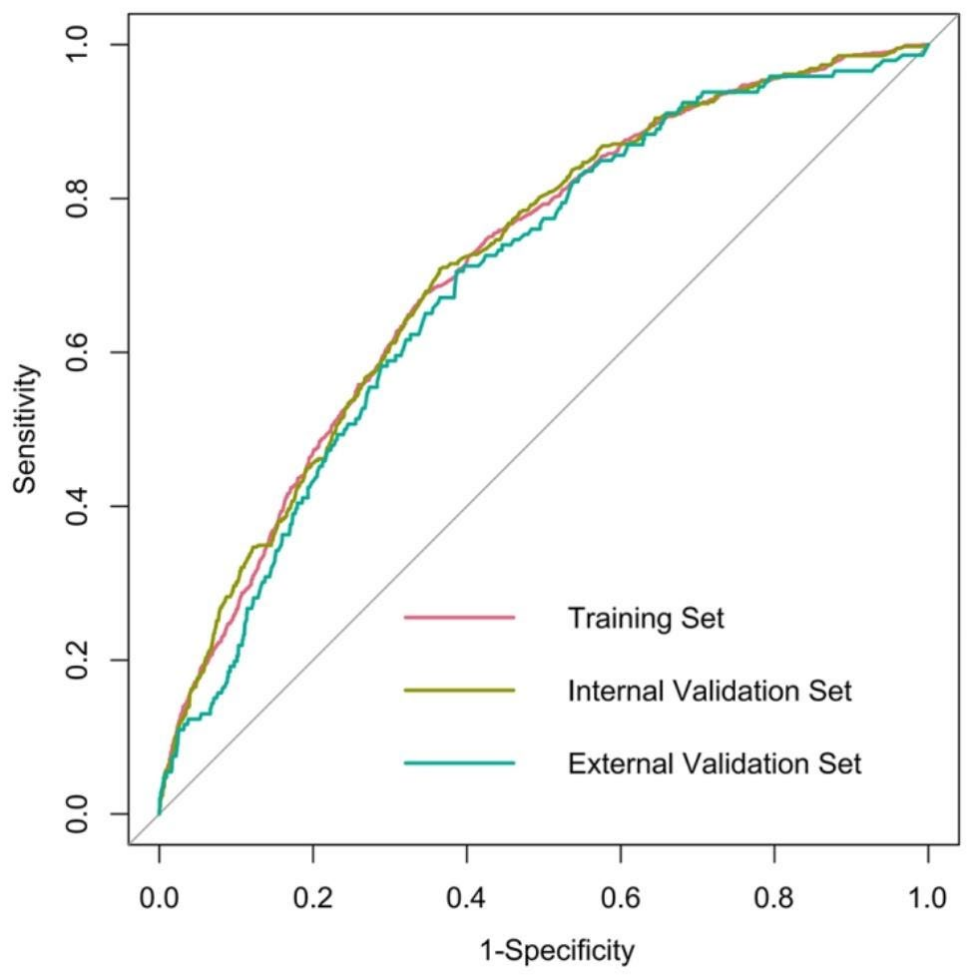
Receiver operating characteristics curves of the model

**Fig. 6:**
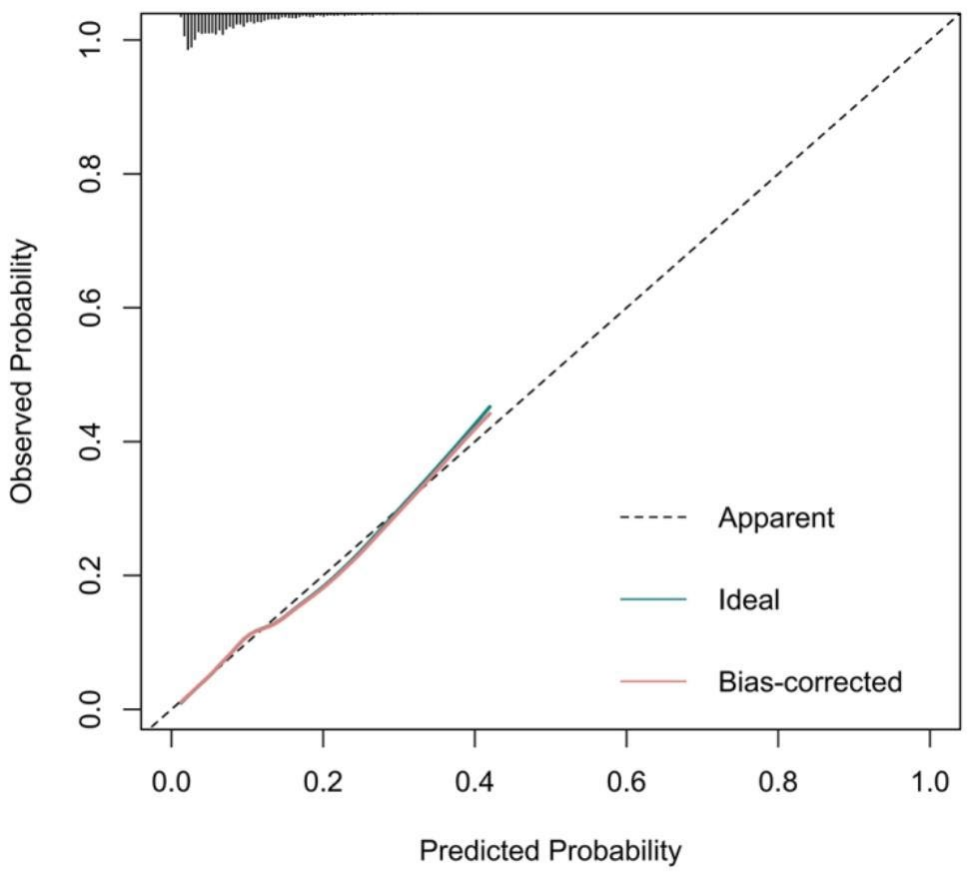
Calibration curves of the model

Figure 4 reveals the prediction model as a nomogram for calculating the probability of OP in elderly patients with T2DM. To use the nomogram, we first drew a line from each parameter value to the score axis, added the scores of all parameters, and finally drew a line from the total score axis to determine the probability of OP in elderly patients with T2DM. The model displayed a high predictive ability, with an AUROC of 0.713 (95% confidence interval [CI]: 0.697–0.730) in the training set (Figs. 5), 0.716 (95% CI: 0.691–0.740) in the internal set, and 0.694 (95% CI: 0.653–0.735) in the external set. The optimal decision probability cut-off value was 0.075. The calibration curve (bootstraps = 1,000) indicated good calibration (Fig. 6). **Supplementary figures S2-S3** respectively revealed calibration curves for the internal and external validation sets. Table 4 presents the detailed performance metrics for the three datasets.

**Table 4:** Detailed performance metrics of the three models

Models	Sensitivity	Specifificity	AUC
	(95%CI)	(95%CI)	(95%CI)
Training set	0.675	0.655	0.713
	0.645–0.706	0.646–0.664	0.697–0.730
Internal validation set	0.708	0.635	0.716
	0.665–0.752	0.622–0.649	0.691–0.740
External validation set	0.705	0.613	0.694
	0.632–0.779	0.597–0.630	0.653–0.735

*AUC: area under the curve; CI: Confidence Interval.*

### Clinical utility of the nomogram prediction model

**Fig. 7:**
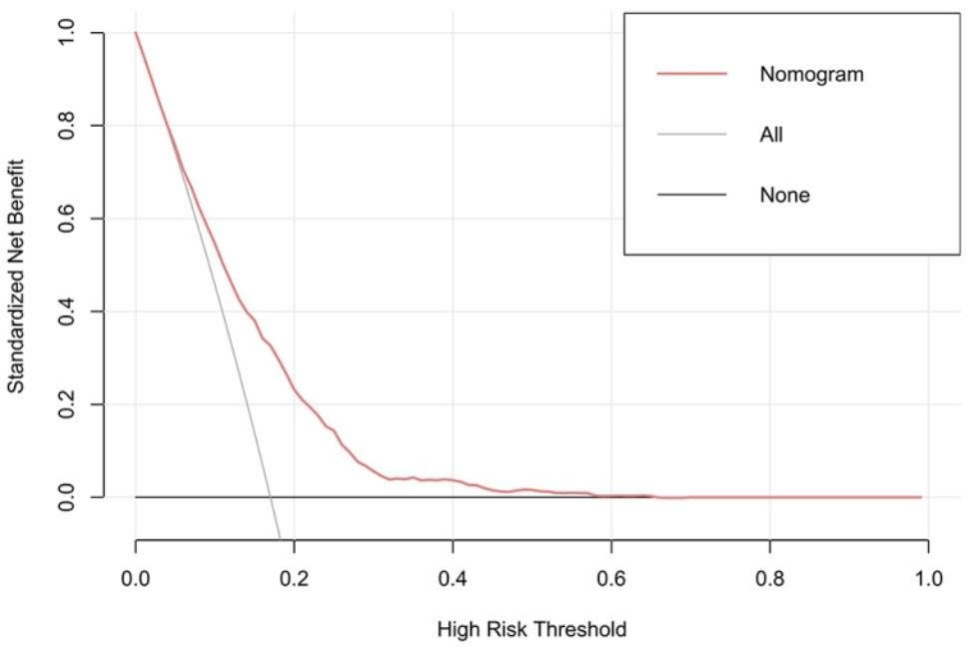
Decision curve analysis of the model. X-axis indicates the threshold probability for OP and Y-axis indicates the net benefit

**Fig. 8:**
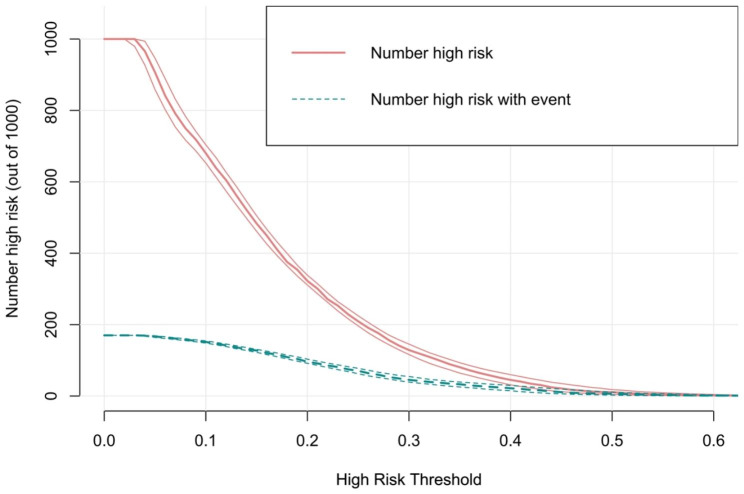
Clinical impact curve of the model. The red curve (number of high-risk individuals) indicates the number of people who are classified as positive (high risk) by the model at each threshold probability; the green curve (number of high-risk individuals with outcome) is the number of true positives at each threshold probability

The clinical utility of the model was evaluated by DCA (Fig. 7). The results indicate that when the threshold probability ranges from 10 to 40%, the model provides greater net benefits. The CIC for OP in elderly patients with T2DM is depicted in Fig. 8. This curve reveals the estimated number of participants deemed to be at high risk of OP. For example, at a 17% risk threshold, out of 1000 patients screened, approximately 400 were deemed high-risk through model analysis. The DCA of the internal and external validation sets are depicted in **Supplementary figures S4-S5**. The CIC of the internal and external validation sets are displayed in **Supplementary figures S6-S7**.

### Construction of an online interface to easily access the model

**Fig. 9:**
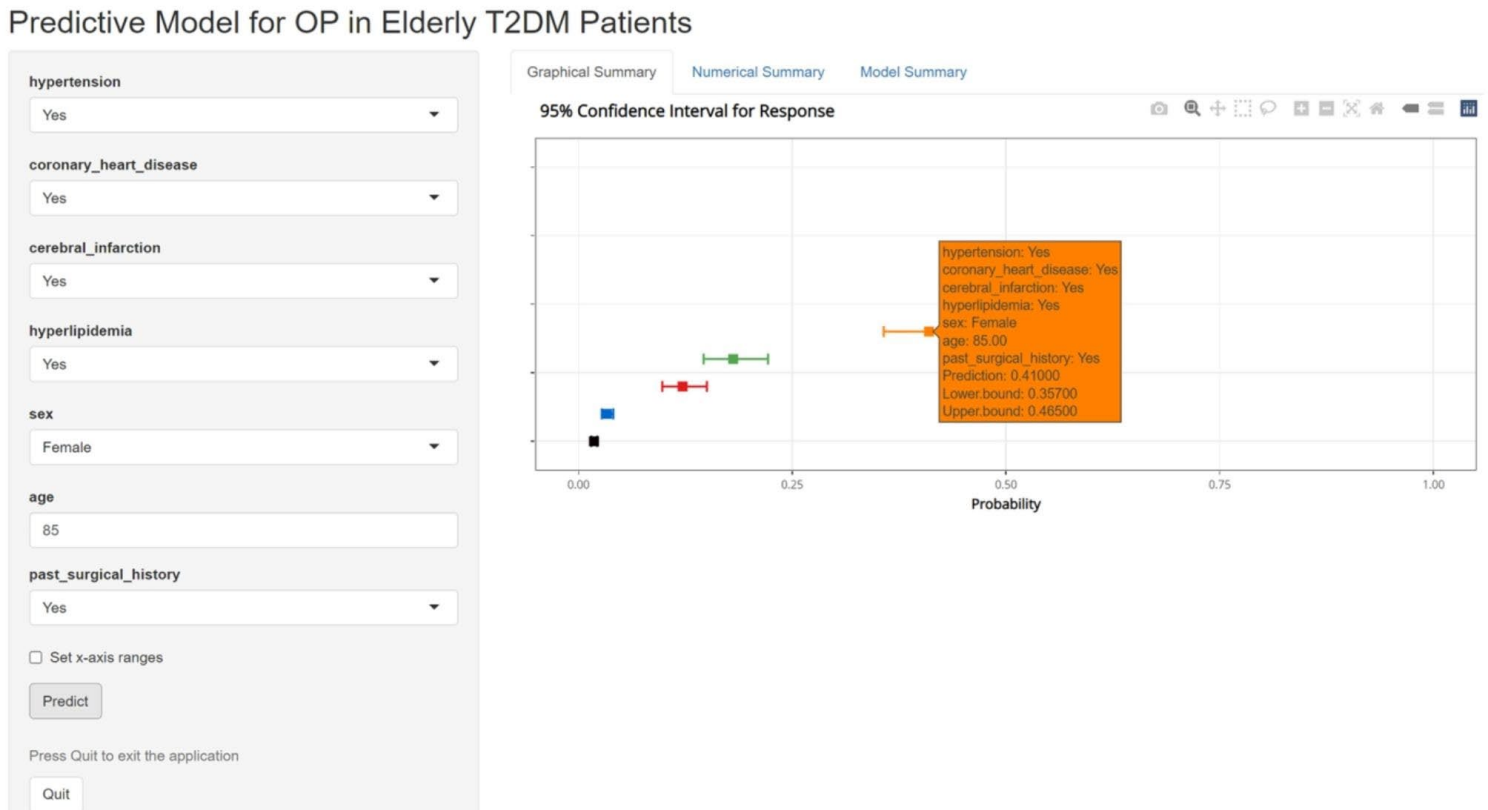
An example of nomogram to predicting OP in elderly patients with T2DM via a link

Finally, we developed a user-friendly interface via a web link (https://juntaotan.shinyapps.io/osteoporosis/) to calculate the precise probability of OP in elderly patients with T2DM. One patient from our study is demonstrated as an example; the likelihood of OP was 0.410 (95% CI: 0.357–0.465) when a female patient aged 85 years had hypertension, CHD, CI, hyperlipidemia, and PSH (Fig. 9).

## Discussion

In this study, we assessed several characteristics and clinical data that may be associated with an increased risk of OP in elderly patients with T2DM. Our study demonstrated that an easy-to-use predictive model based on seven predictors (age, sex, hypertension, CHD, CI, hyperlipidemia, and PSH) could identify underlying OP, with an AUROC of 0.713, specificity of 0.655, and sensitivity of 0.675.

Although there are currently many screening tools for OP, their applicability and effectiveness remain challenging. In a cross-sectional study, 786 Malaysians were recruited to verify the performance of OSTA in identifying subjects with OP, as determined by DXA [22]. The results showed that the sensitivity of OSTA in identifying subjects with suboptimal bone health was only 0.323, with an AUROC of only 0.618. Even after adjusting the cutoff value of OSTA, its specificity in identifying male and female patients only reached 0.555 and 0.614, respectively. In another study, researchers used data from the National Health and Nutrition Examination Survey to validate the effectiveness of MORES in identifying the risk of vertebral OP in men [23]. The results showed that the sensitivity and specificity of MORES were only 0.582 (95% CI: 0.460–0.694) and 0.652 (95% CI: 0.627–0.676), respectively. The study by Crandall et al. also showed that both FRAX and Garvan fracture risk calculator had low specificity in detecting incident hip fracture during the 10-year follow-up (Garvan 0.306 (95% CI: 0.303–0.310) and FRAX 0.431 (95% CI: 0.427–0.435)) [24].

Due to long-term blood sugar fluctuations, T2DM patients may experience metabolic disorders involving three major nutrients (protein, fat, and sugar), which are not conducive to the bone matrix [25]. Additionally, high blood sugar levels can cause osmotic diuresis, resulting in a significant loss of trace elements such as calcium and phosphorus, thereby leading to a decrease in bone density [26]. Therefore, T2DM patients have a higher risk of developing OP than others. In this study, we established that older age is a risk factor for T2DM patients with OP. With an increase in age, T2DM patients have a decrease in their immune system and hormone levels. Moreover, they are prone to disorders in calcium and phosphorus metabolism, decreased osteocalcin levels, and decreased bone remodeling function, which increases the probability of OP occurrence [27].

Several studies have confirmed that sex is an important risk factor for OP [28–30]. Here, we found that female patients with T2DM had a higher risk of OP than male patients (OR = 3.138, 95% CI: 2.668–3.692; P < 0.001). In postmenopausal women, estrogen levels and osteoblast activity decreases while osteoclast activity increases. This in turn leads to bone loss and decreased bone density, resulting in OP. In the male population, testosterone decrease may have a similar but less significant impact, with sex being the strongest influencing factor of OP occurrence [31]. Martin et al. showed that halving estrogen concentration would reduce bone mineral density of the lumbar vertebrae by 10% and the femoral neck by 12% [32]. Therefore, the elderly female population should appropriately consume calcium-containing foods, including shrimp skin, fish, milk, and dairy products, to supplement nutrition, and maintain bone density and metabolic balance, thereby preventing OP.

The traditional concept indicates OP is purely a metabolic bone disease. However, accumulating evidence suggests that OP may be regarded as a risk factor for cardiovascular disease, similar to other traditional risk factors (e.g., hypertension, CI, CHD, hyperlipidemia, and diabetes) [33–35]. This represents a paradigm shift in the prospects of OP. OP and cardiovascular diseases have similar risk factors, including diabetes, smoking, excessive drinking, a sedentary lifestyle, aging, and dyslipidemia. This may partially explain the association between OP and cardiovascular disease. The results of this study suggest that hypertension, CI, hyperlipidemia, and CHD are risk factors for OP in elderly patients with T2DM. Consistent with our research results, a survey of the health and nutrition of Korean residents showed that OP in the femoral neck was significantly associated with hypertension (OR = 1.422, 95% CI: 1.107–1.827; P = 0.006) [36]. The mechanism by which hypertension causes OP may be that the RAAS system not only plays an important role in hypertension, but also that angiotensin is a factor regulating osteoclast bone absorption [37]. In addition, OP may be associated with abnormal calcium metabolism and hypertension-related bone loss. Hu et al. stated that hypertension, CHD, and CI were the main risk factors for OP in the elderly [38]. The incidence rates of OP in the two-vessel and three-vessel disease groups were significantly higher than those in the single-vessel disease group. Furthermore, this study suggests that PSH is an important risk factor for OP in elderly patients with T2DM (OR = 1.384, 95% CI: 1.201–1.594). Previous studies confirmed that gastrotomy and cervical disc arthroplasty [39–42] may easily lead to OP. Therefore, for elderly T2DM patients with PSH, systematic recovery of bone mineral density is necessary.

The advantages of this study mainly are two-fold: first, the use of a large sample and multicenter data to construct the prediction model; second, the variables used to construct the predictive model are simple and easy to obtain, which greatly improves the model’s generalizability and facilitates its application to clinical practice. However, our study has some limitations. First, it was a retrospective study. Retrospective studies provide weaker evidence compared with prospective studies. Hence, the interpretation of these findings should be considered with caution. Second, although our study evaluated the demographic characteristics and baseline clinical data of patients, it may be advantageous to identify the predictors of OP in elderly patients with T2DM and improve the predictive performance of the model by evaluating other variables, such as disability and use of drugs and omics data. Therefore, further studies with complete data on all the pertinent covariates would be useful.

## Conclusions

In a large retrospective study of elderly patients with T2DM admitted to six tertiary hospitals in Southwest China, we observed that the key factors influencing OP were age, sex, hypertension, CHD, CI, hyperlipidemia, and PSH. Hence, the primary management step should focus on optimizing the influencing factors to reduce the risk of OP in elderly patients with T2DM. Additionally, our study suggests that a simple predictive model may be used as an automatic screening tool to provide additional reference values for the priority identification of high-risk patients.

### Electronic supplementary material

Below is the link to the electronic supplementary material.

Supplementary Material 1

## Data Availability

Data supporting the results of this study can be obtained on request to the authors. Yongjun Hu. (Email: lionhu@sina.com) should be contacted if someone wants to request the data from this study.

## Code Availability

An online interface (https://juntaotan.shinyapps.io/osteoporosis/) was developed to provide convenient access for users.
