# Supplementary material for: Development and validation of a risk prediction model for osteoporosis in elderly patients with type 2 diabetes mellitus: a retrospective and multicenter study
Source: BMC Geriatr. 2023 Oct 27;23:698. doi: 10.1186/s12877-023-04306-1 (PMC10604807; doi:10.1186/s12877-023-04306-1)
Supplement: Supplementary file 1 — Supplementary Material 1 [file 12877_2023_4306_MOESM1_ESM.docx]

**Supplementary information**

**Figure S1. Patient flow diagram**

**
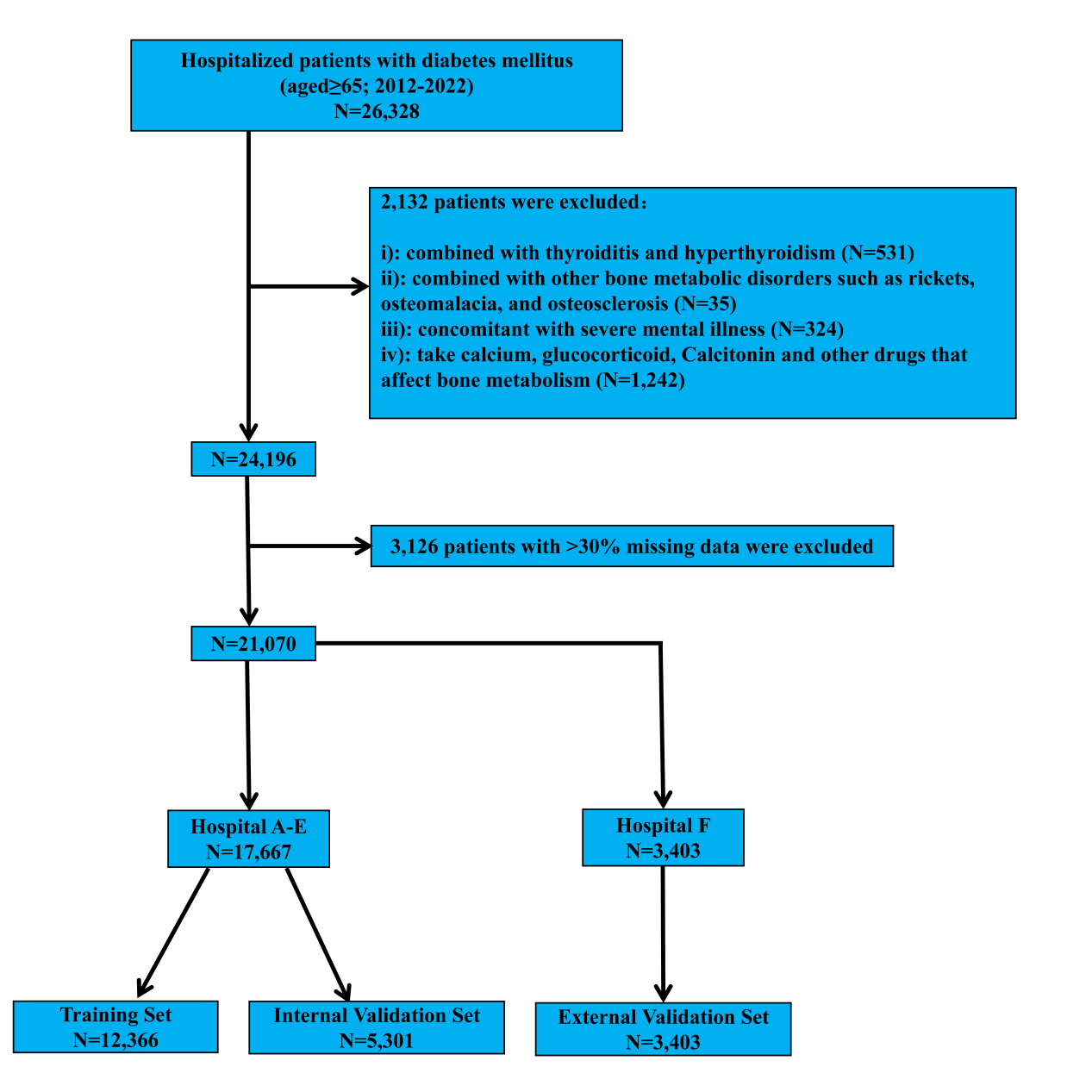
**

**Table S1 Comparison of continuous variables in external validation set before and after multiple imputation**

| Variables | Before interpolation | After interpolation | *P* values |
| --- | --- | --- | --- |
| SBP(IQR, mmHg) | 140.00(127.00,155.00) | 140.00(127.00,155.00) | 0.852 |
| DBP(IQR, mmHg) | 78.00(70.00,87.00) | 78.00(70.00,87.00) | 0.670 |
| pulse(IQR, bpm) | 81.00(73.00,92.00) | 81.00(73.00,92.00) | 0.761 |
| AST(IQR, IU/L) | 19.76(15.85,25.59) | 19.80(15.85,25.70) | 0.774 |
| ALT(IQR, IU/L) | 19.20(14.08,27.34) | 19.19(14.04,27.50) | 0.957 |
| TGs(IQR, mmol/l) | 1.44(1.05,2.09) | 1.41(1.03,2.05) | 0.165 |
| NLR(IQR) | 3.10(2.13,5.02) | 3.11(2.14,5.12) | 0.554 |
| PLR(IQR) | 131.29(97.68,182.27) | 131.77(97.76,183.61) | 0.796 |
| LMR(IQR) | 3.20(2.20,4.47) | 3.15(2.15,4.43) | 0.248 |
| NPAR(IQR, ml/g) | 16.15(14.13,18.80) | 16.20(14.13,18.88) | 0.773 |
| CREA(IQR, umol/l) | 72.36(59.50,90.76) | 72.40(59.49,90.92) | 0.916 |
| UA(IQR, umol/l) | 321.60(262.22,393.44) | 321.81(262.21,393.97) | 0.909 |
| LDL-C(IQR, mmol/l) | 2.49(1.90,3.15) | 2.48(1.90,3.12) | 0.630 |
| HDL-C(IQR, mmol/l) | 1.19(1.00,1.43) | 1.20(1.00,1.44) | 0.509 |
| HbA1c(IQR, %) | 7.50(6.60,9.00) | 7.50(6.60,9.00) | 0.907 |
| GFR(IQR, mL/min) | 85.01(67.02,103.64) | 84.95(66.80,103.58) | 0.928 |

*SBP: systolic blood pressure; DBP: diastolic blood pressure; AST:aspartate aminotransferase; ALT: alanine aminotransferase; TGs: triglycerides; NLR: neutrophil-to-lymphocyte ratio; PLR: platelet-to-lymphocyte ratio; LMR: lymphocyte to monocyte ratio; NPAR: neutrophil percentage-to-albumin ratio; CREA: creatinine; UA: uric acid; LDL-C: low density lipoprotein cholesterol; HDL-C: high density lipoprotein cholesterol; HbA1c: glycated hemoglobin; GFR: glomerular filtration rate; IQR: interquartile range.*

**Figure S2. Calibration curves of the model in internal validation set**

**
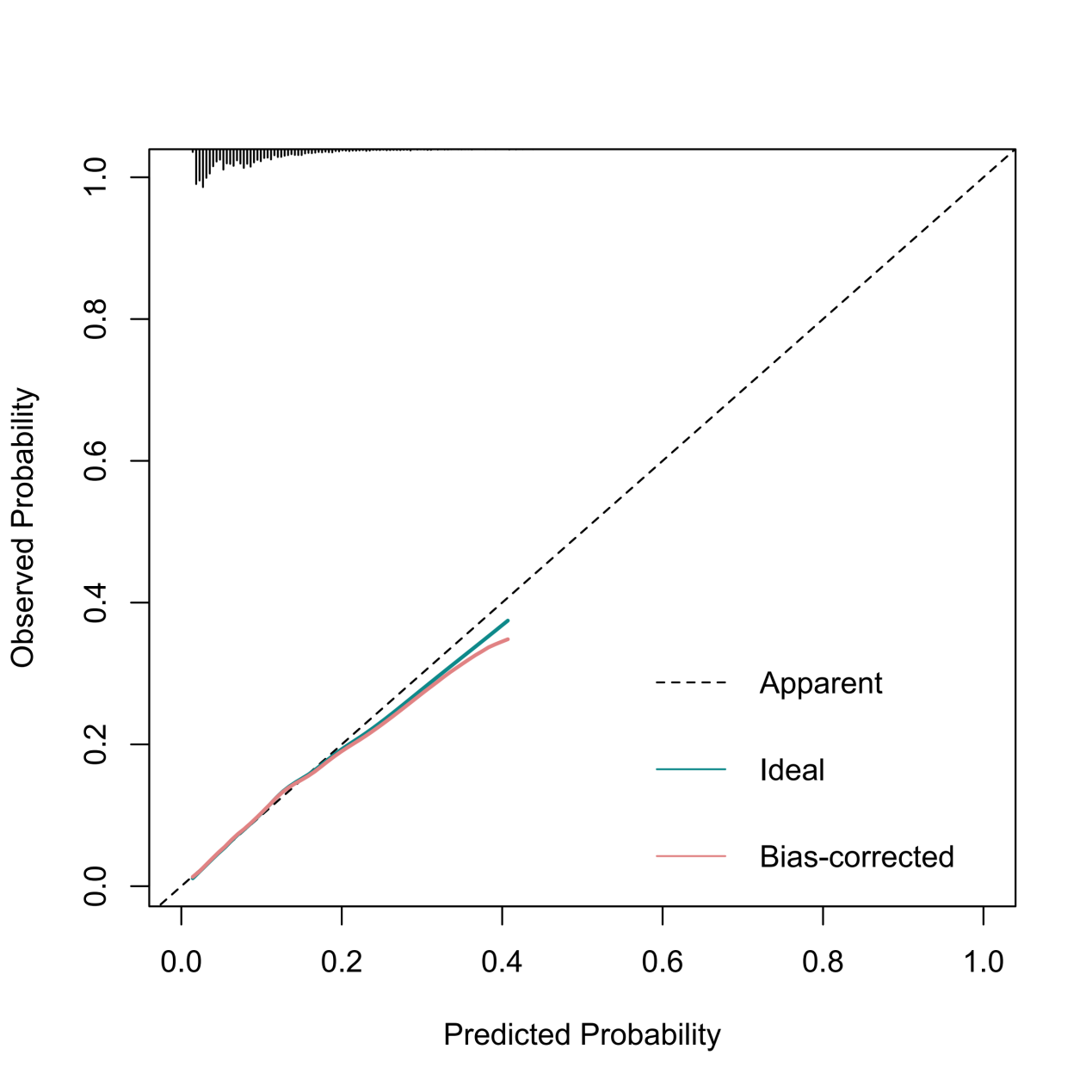
**

**Figure S3. Calibration curves of the model in external validation set**

**
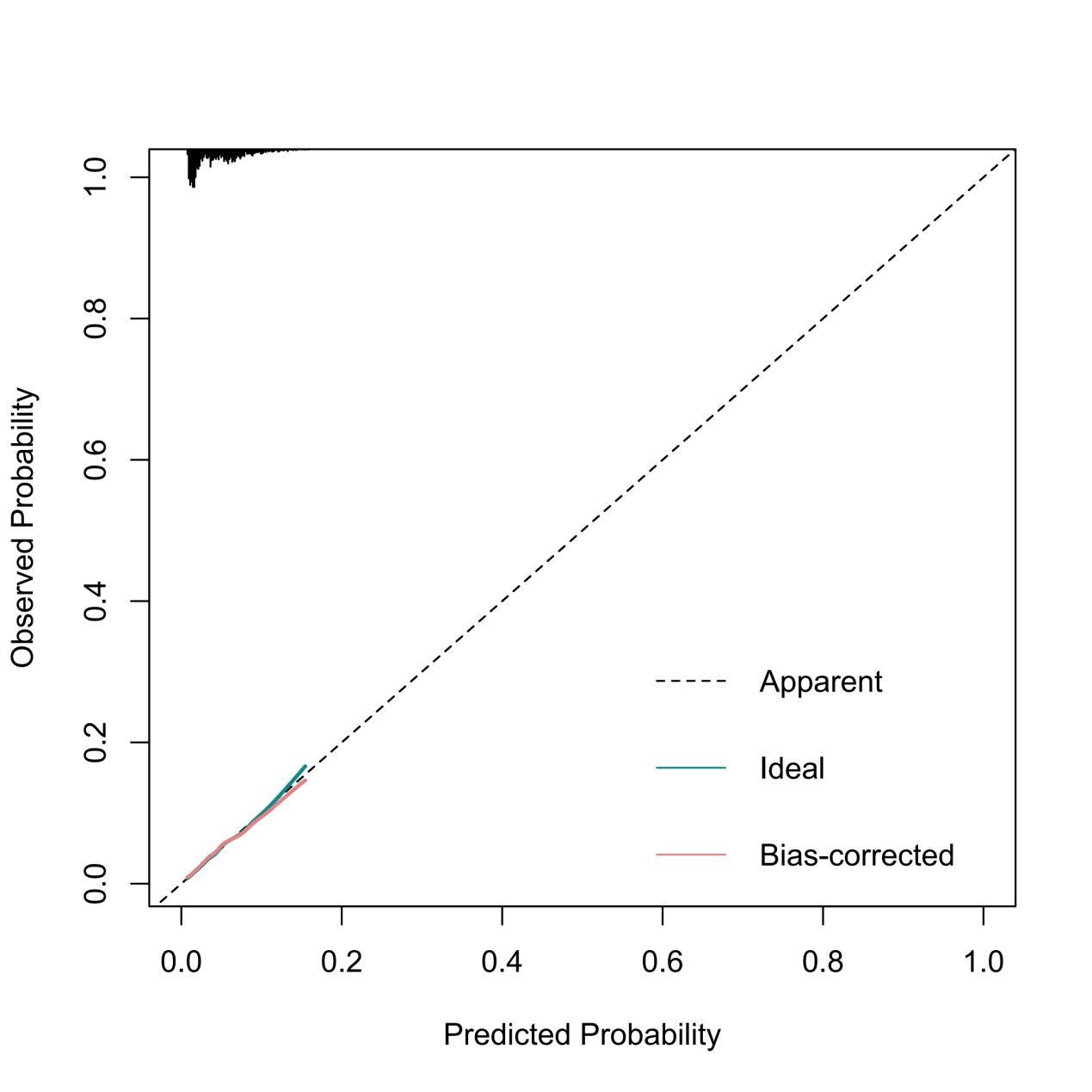
**

**Figure S4. DCA of the nomogram in internal validation set**

**
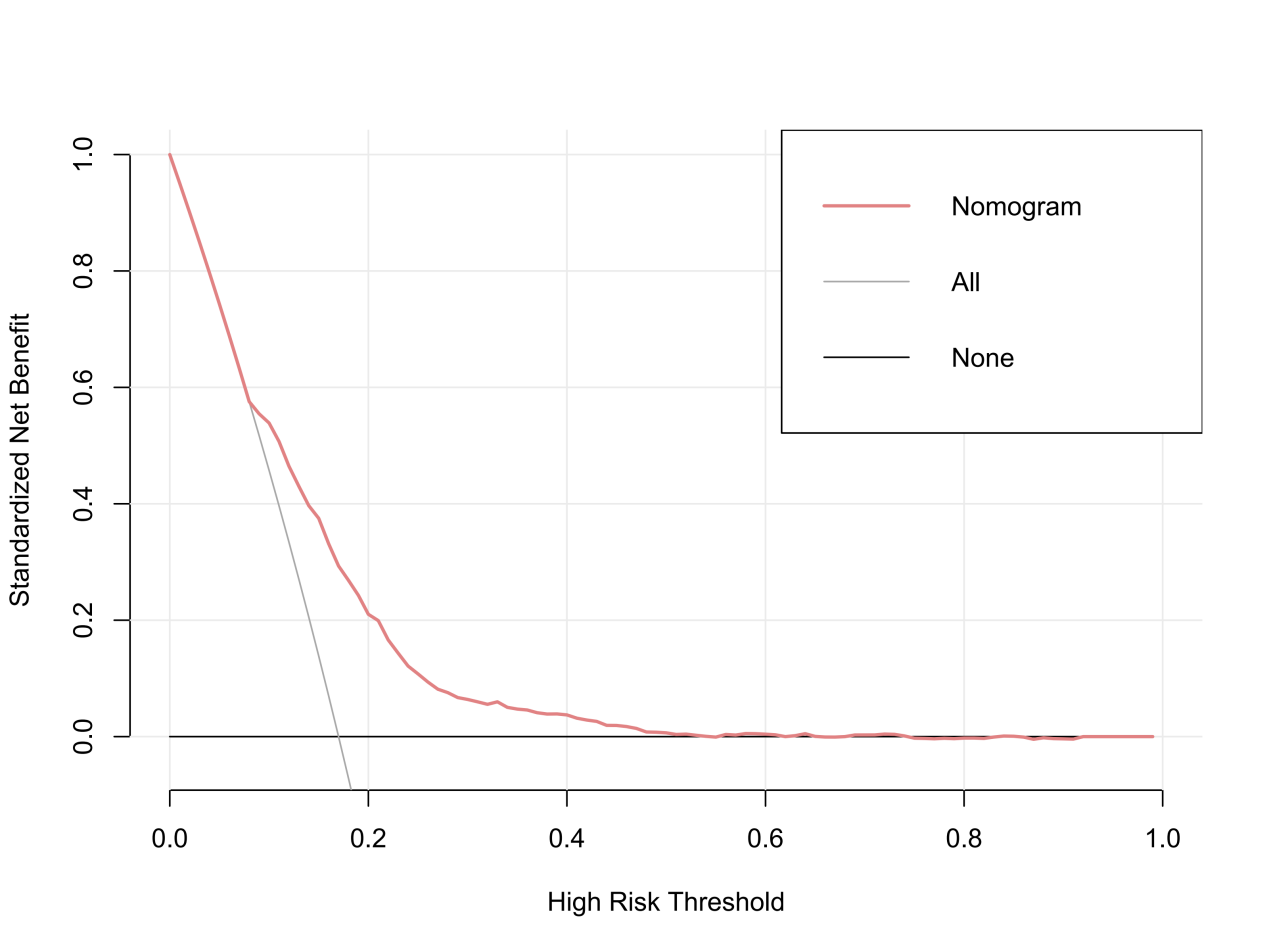
**

**Figure S5. DCA of the nomogram in external validation set**

**
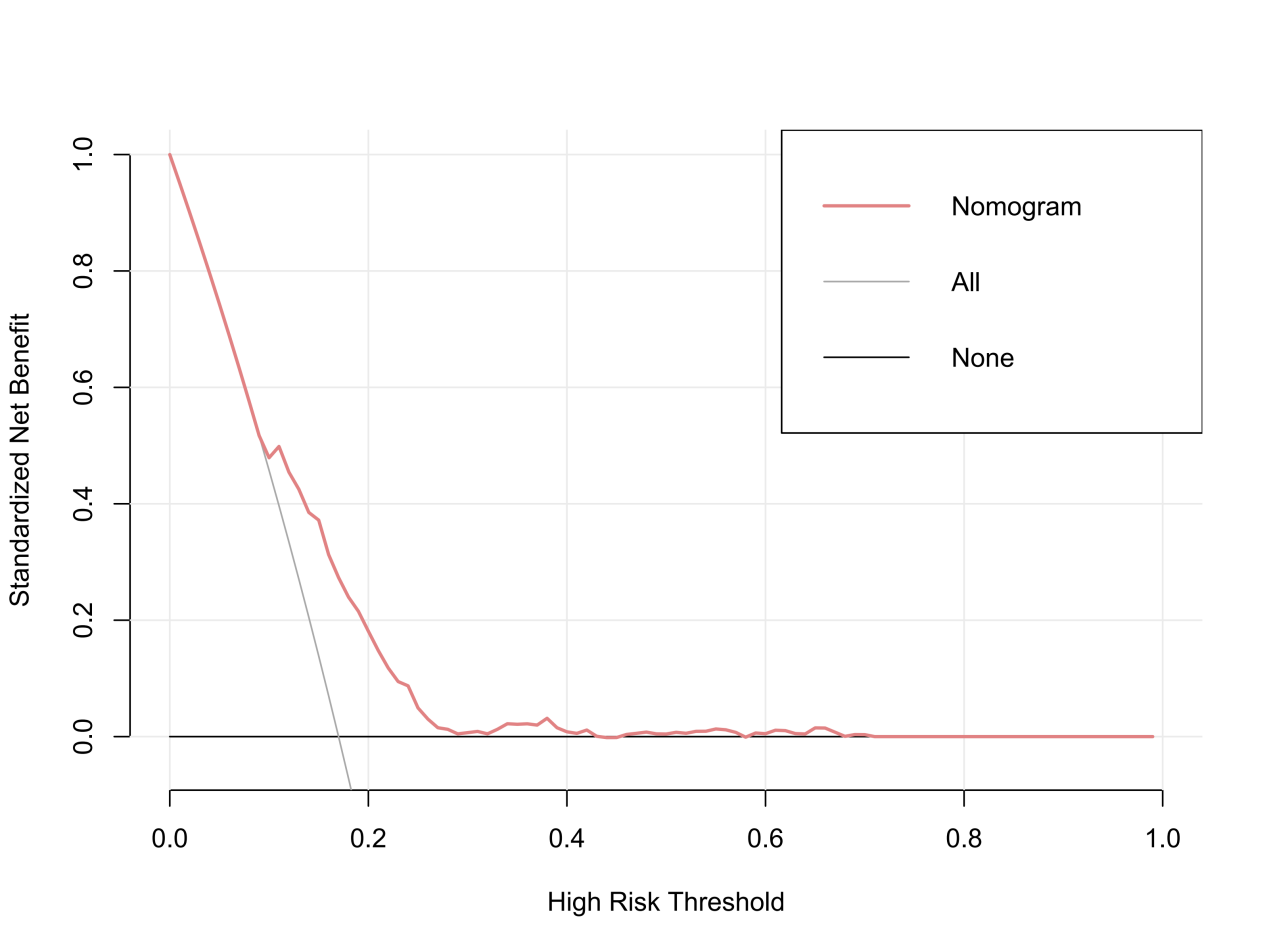
**

**Figure S6. CIC of the nomogram in internal validation set**

**
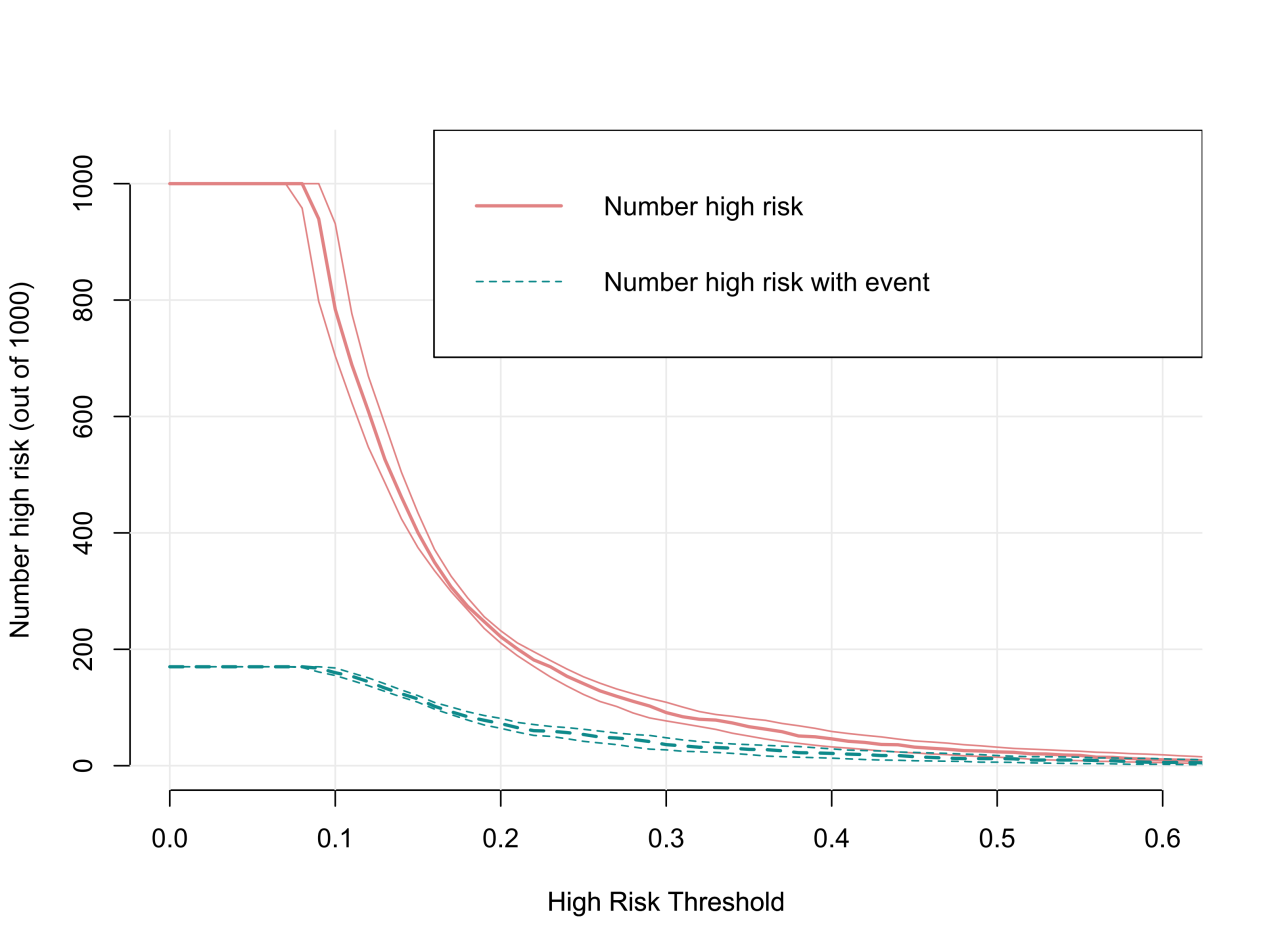
**

**Figure S7. CIC of the nomogram in external validation set**

**
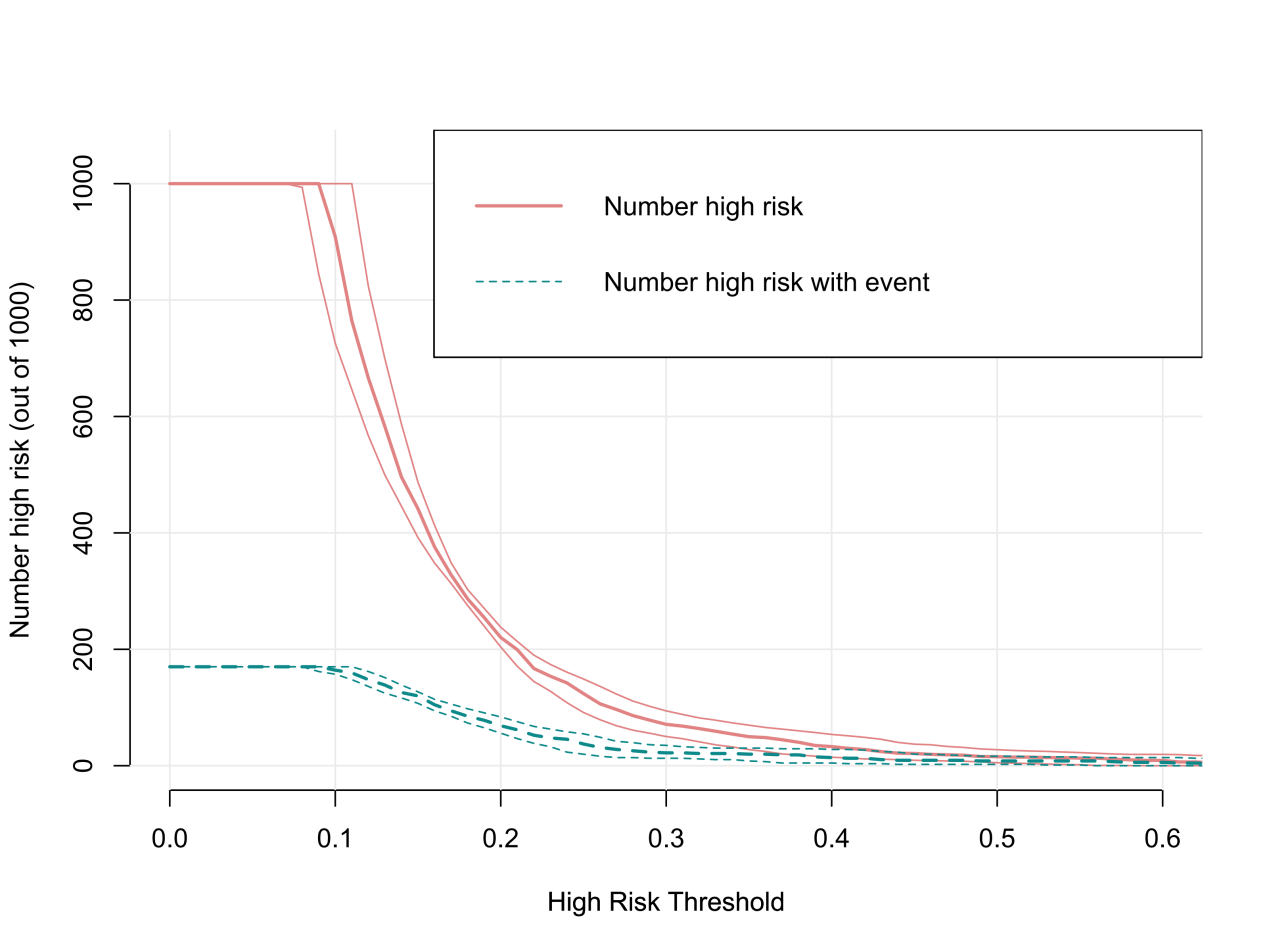
**
